# Supplementary material for: Lipid Profile of Larix cajanderi Mayr in Adaptation to Natural Conditions in the Cryolithozone
Source: Int J Mol Sci. 2024 Dec 28;26(1):164. doi: 10.3390/ijms26010164 (PMC11719822; doi:10.3390/ijms26010164)
Supplement: Supplementary file 1 [file ijms-26-00164-s001.zip › Figure S1.pdf]

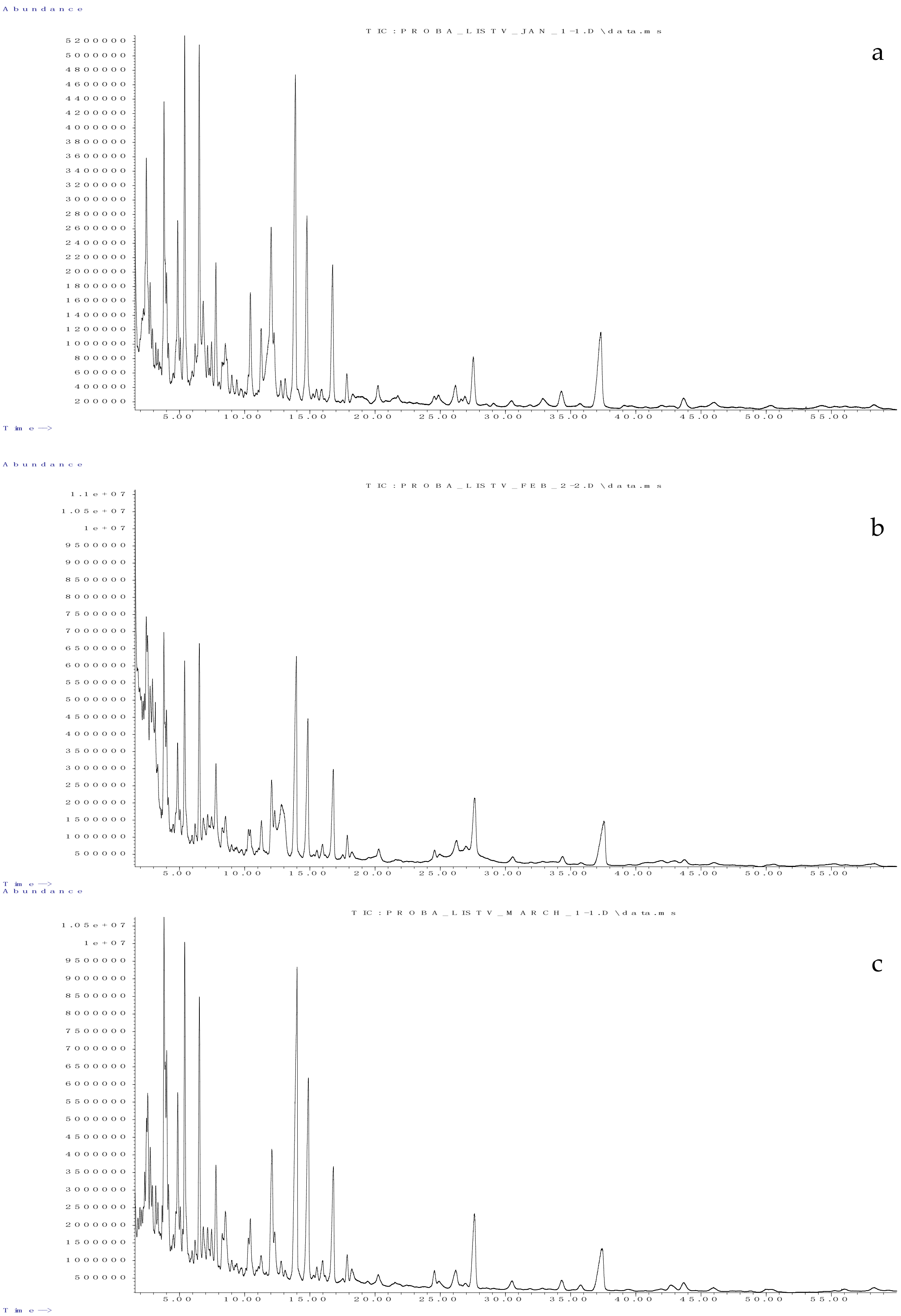
Abundance
TIC: PROBA_LISTV_JAN_1-1.D\data.ms
a
Time-->
Abundance
TIC: PROBA_LISTV_FEB_2-2.D\data.ms
b
Time-->
Abundance
TIC: PROBA_LISTV_MARCH_1-1.D\data.ms
c
Time-->

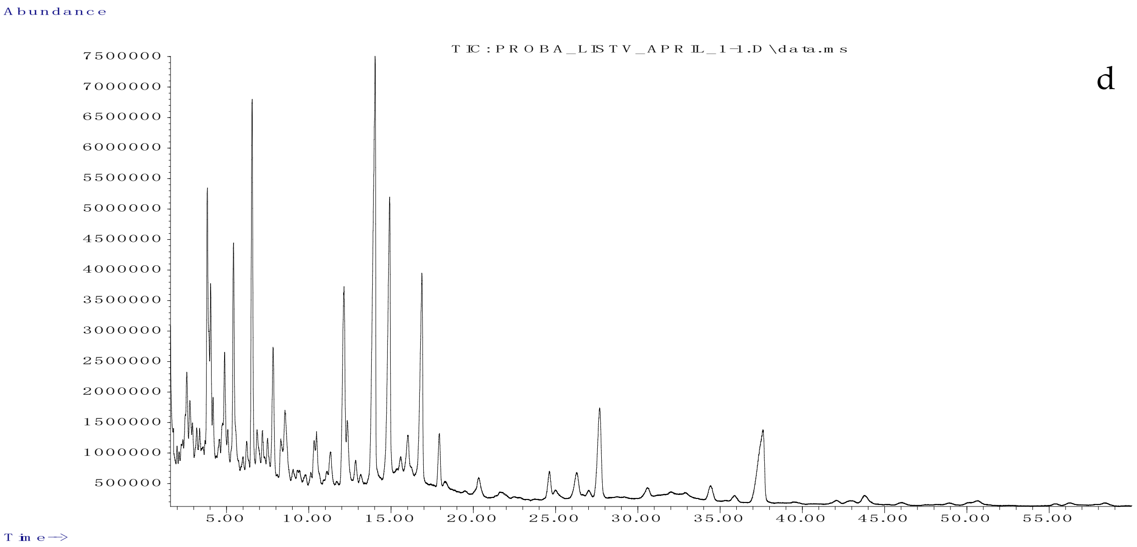
Abundance
TIC: PROBA_LISTV_APRIL_1-1.D\data.ms
d
7500000
7000000
6500000
6000000
5500000
5000000
4500000
4000000
3500000
3000000
2500000
2000000
1500000
1000000
500000
5.00
10.00
15.00
20.00
25.00
30.00
35.00
40.00
45.00
50.00
55.00
Time-->

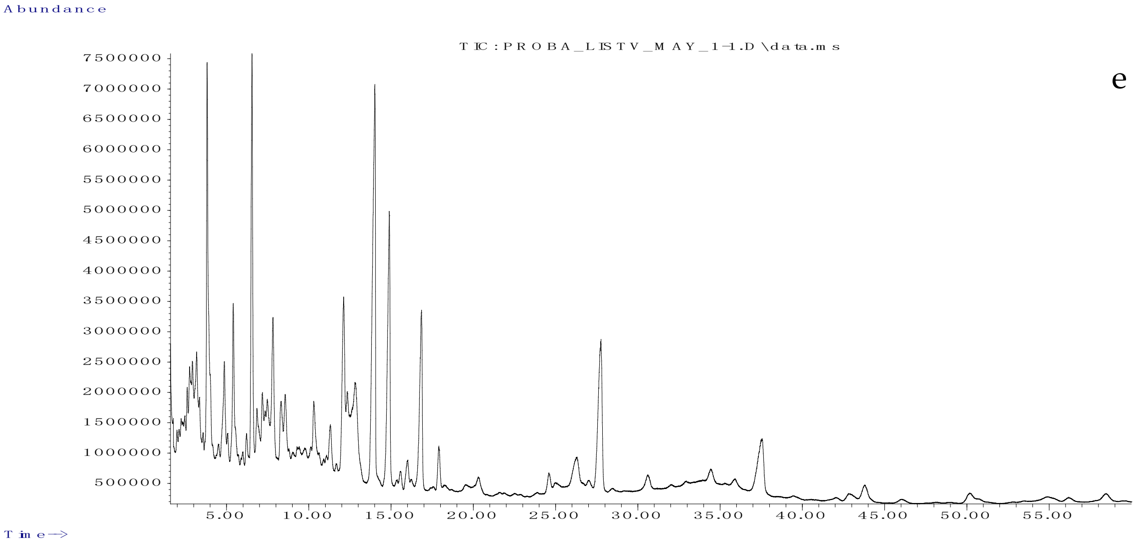
Abundance
TIC: PROBA_LISTV_MAY_1-1.D\data.ms
e
7500000
7000000
6500000
6000000
5500000
5000000
4500000
4000000
3500000
3000000
2500000
2000000
1500000
1000000
500000
5.00
10.00
15.00
20.00
25.00
30.00
35.00
40.00
45.00
50.00
55.00
Time-->

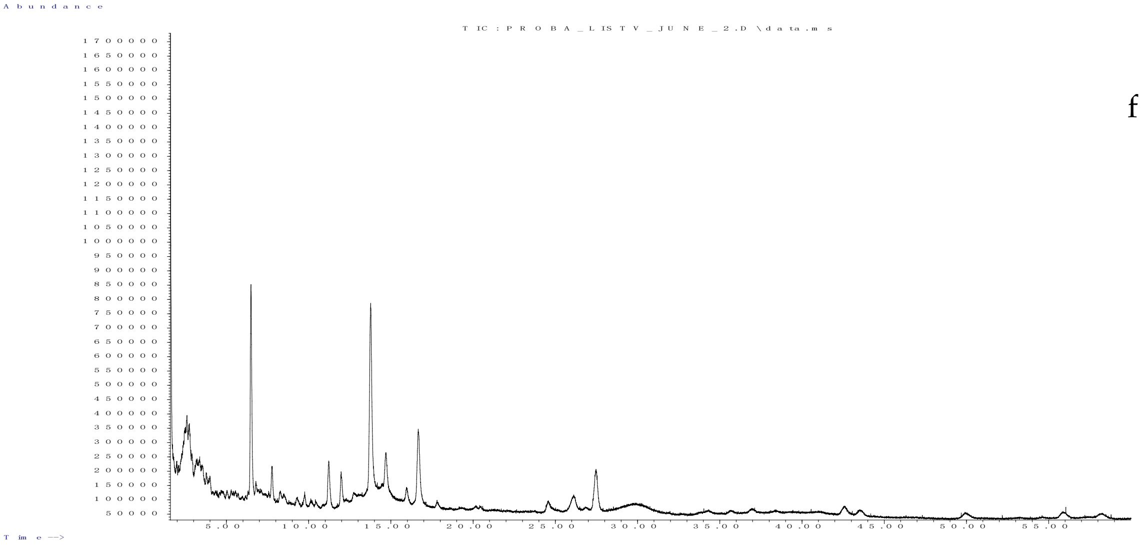
Abundance
TIC: PROBA_LISTV_JUNE_2.D\data.ms
f
1700000
1650000
1600000
1550000
1500000
1450000
1400000
1350000
1300000
1250000
1200000
1150000
1100000
1050000
1000000
950000
900000
850000
800000
750000
700000
650000
600000
550000
500000
450000
400000
350000
300000
250000
200000
150000
100000
50000
5.00
10.00
15.00
20.00
25.00
30.00
35.00
40.00
45.00
50.00
55.00
Time-->

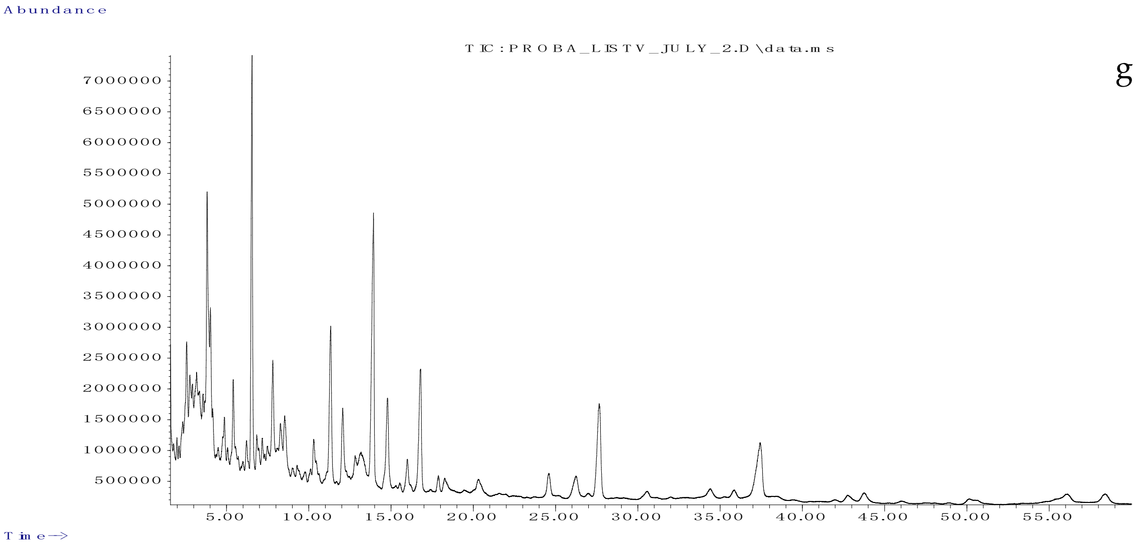
Abundance
TIC: PROBA_LISTV_JULY_2.D\data.ms
g
Time-->

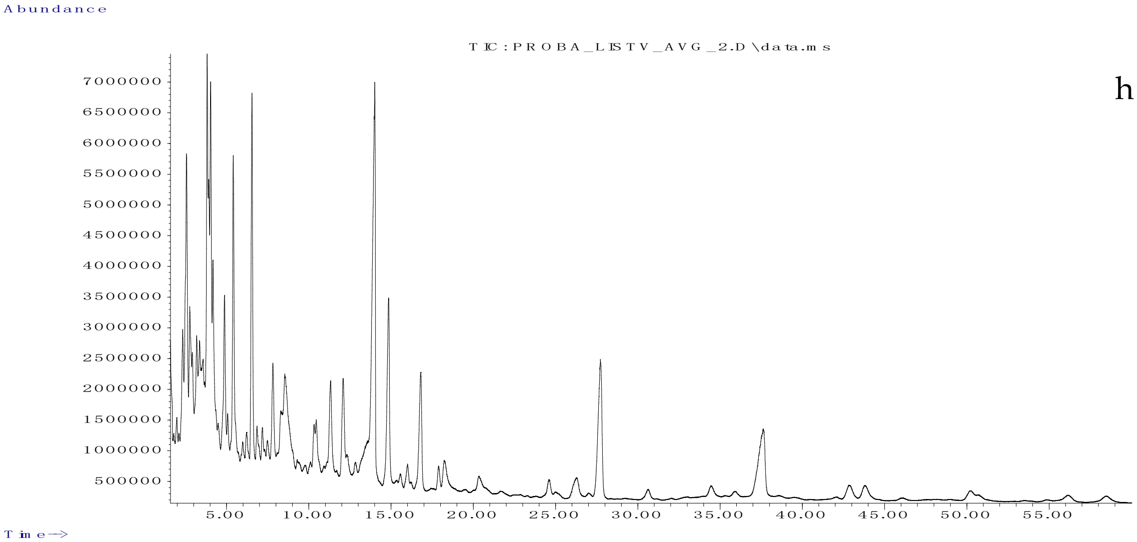
Abundance
TIC: PROBA_LISTV_AVG_2.D\data.ms
h
Time-->

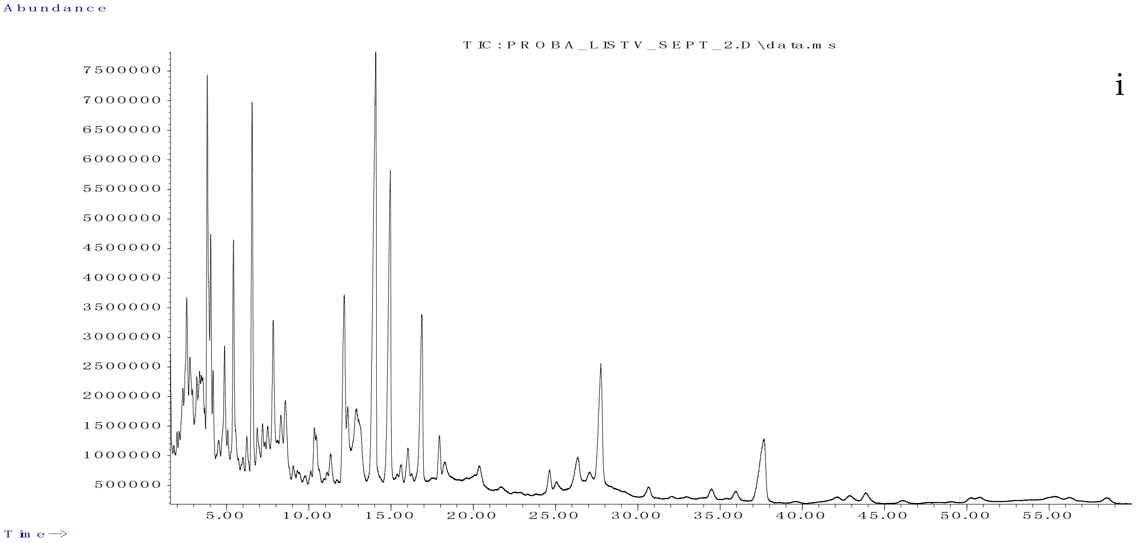
Abundance
TIC: PROBA_LISTV_SEPT_2.D\data.ms
i
Time-->

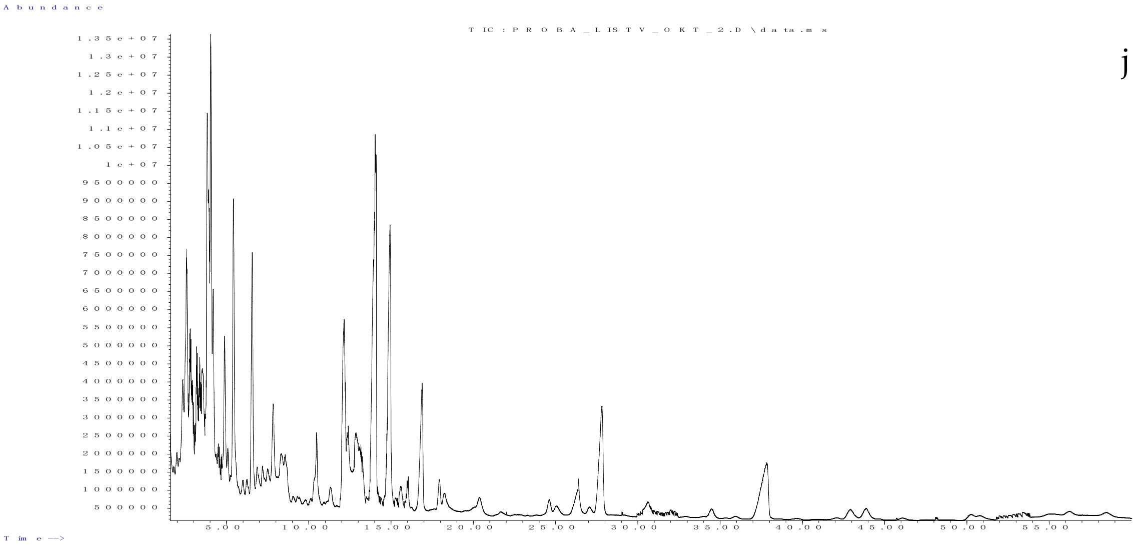

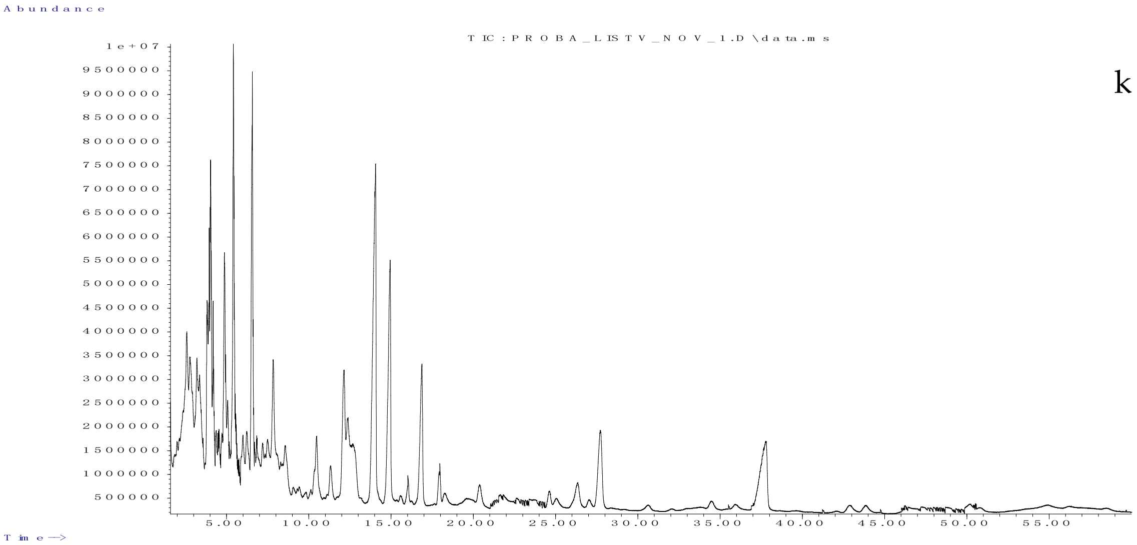

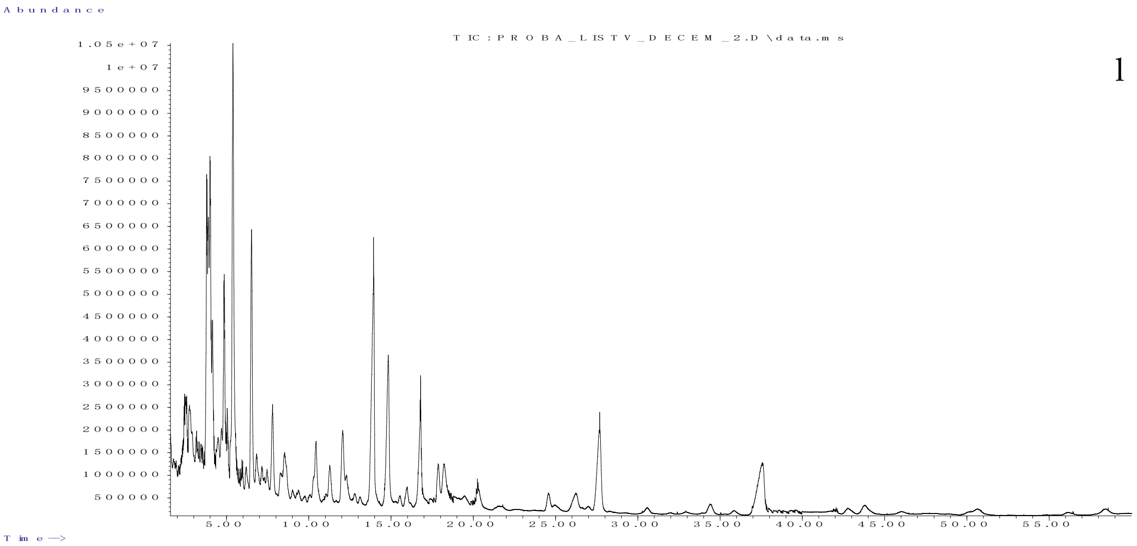

Figure S1. Examples of chromatograms of FAME in the shoots of *L. cajanderi* in a one-year cycle. January (**a**), February (**b**), March (**c**), April (**d**), May (**e**), June (**f**), July (**g**), August (**h**), September (**i**), October (**j**), November (**k**), December (**l**).
